# PatchWorkPlot: simultaneous visualization of local alignments across multiple sequences

**DOI:** 10.1093/bioinformatics/btaf504

**Published:** 2025-09-15

**Authors:** Mariia Pospelova, Yana Safonova

**Affiliations:** Computer Science and Engineering Department, Pennsylvania State University, State College, PA 16802, United States; Computer Science and Engineering Department, Pennsylvania State University, State College, PA 16802, United States; Huck Institutes of Life Science, Pennsylvania State University, State College, PA 16802, United States

## Abstract

**Motivation:**

Revealing structural variations within and across populations is crucial for understanding their diversification mechanisms and roles. Existing tools for visualization of structural variations often require labor-intensive figure preparation and are limited in their ability to integrate annotations.

**Results:**

We developed PatchWorkPlot, a tool for automated visualization of pairwise alignments of multiple annotated sequences as dot plots combined into a single matrix. PatchWorkPlot enables exploration of positions, breakpoints, and architectures of structural variations across two or more sequences. The tool supports customization of visualization parameters and produces high-resolution, publication-ready figures. PatchWorkPlot significantly reduces manual work and simplifies the generation of complex plots for various cases, from individual loci to large-scale comparative projects.

**Availability and implementation:**

PatchWorkPlot is implemented using Python 3 and is publicly available at GitHub: github.com/yana-safonova/PatchWorkPlot.

## 1 Introduction

Development of sequencing technologies and genome assembly methods has enabled reconstruction of previously inaccessible and repetitive genomic regions (Nurk *et al.* 2022, Makova *et al.* 2024, Yoo *et al.* 2025). Analysis of structural variations in such sequences across closely related subjects or species is an important step in understanding their role and diversification mechanisms. A dot plot representing all local alignments between two sequences remains the main tool for initial visual assessment of sequence similarities and structures (Krumsiek *et al.* 2007, Sweeten *et al.* 2024). A dot plot of two sequences, *S*_1_ and *S*_2_ is a collection of line segments corresponding to all local alignments. A line segment representing an alignment of substrings of lengths *l*_1_ and *l*_2_ starts at (*x*, *y*) and extends to (*x*+ *l*_1_, *y*+ *l*_2_), where *x* and *y* are starting alignment positions in *S*_1_ and *S*_2_, respectively.

Availability of genomes from closely related species and population-wide data for the same species raises a question about a convenient way to combine results of local alignments of more than two sequences. While linear genome diagrams (e.g. SyRI by Goel *et al.* 2019) might be a tool of choice for non-repetitive sequences, it is less informative for highly repetitive sequences. To overcome this challenge, we present PatchWorkPlot, a tool that analyzes multiple sequences, computes their pairwise alignments using sensitive local alignment tools, and visualizes the results in a matrix format for intuitive assessment. PatchWorkPlot produces high-quality, ready-to-publish figures and has a convenient interface for fine-tuning the results.

## 2 Methods

### 2.1 Software description

PatchWorkPlot includes two main steps: computing alignments and visualizing them as dot plots. Alignment is performed using an existing tool, and the current version supports LastZ (Harris 2007), minimap2 (Li 2018), and MashMap (Jain *et al.* 2018). Once alignments are computed, PatchWorkPlot considers the orientation of the first sequence as the main one and orients other sequences with respect to it. If the longest alignments to the first sequence are in reverse orientation, the corresponding sequence will be reversed. PatchWorkPlot then generates dot plots for all sequence pairs and incorporates annotations if provided. All pairwise alignments are saved, allowing the tool to reuse them when rerun on the same output directory, significantly reducing the total running time.

### 2.2 Input and output files

PatchWorkPlot takes a configuration file in the .CSV/.TSV formats as an input. Each input sequence is described as a line in the configuration file. The configuration file has three mandatory columns (“Fasta”: paths to sequences in the FASTA format, “SampleID”: unique sequence IDs, “Label”: sequence labels that will be shown in the final plot) and two optional columns (“Annotation”: paths to annotations in the BED format, “Strand”: orientations of the input sequences). If orientations are provided, PatchWork plot will use them; otherwise, it will determine them automatically as described above. PatchWorkPlot also works with a simplified input where all sequences are combined into a single FASTA, unique identifiers are shown as headers, and all annotations are combined into a single BED file. PatchWorkPlot reports pairwise dot plots combined into a triangular matrix as well as individual pairwise dot plots as PNG and PDF files.

### 2.3 Visualization routine

PatchWorkPlot visualizes pairwise dot plots and arranges them as an upper (lower) triangular matrix, where a cell (*i*, *j*) shows a dot plot corresponding to alignments of the *i*-th and *j*-th input sequences if *i *≤* j* (*i *≥* j*). Dot plots on the main diagonal correspond to self-alignments of the input sequences. Each alignment passing the minimum length threshold (“—min-len,” default: 5 kbp) is shown as a colored line segment. PatchWorkPlot determines the alignment color based on its percent identity (PI), provided it falls within the range set by the “—min-pi” and “—max-pi” parameters (default: 85% and 100%). If the PI value is outside this range, the closest boundary value is used instead. It then projects the PI value into a Python colormap (default: “rainbow”) to assign the color. A different Python colormap or a single color can be specified via “—cmap” or “—color” options, respectively. A user can also change the orientation of the colormap (“—reverse,” default: True) and the width of alignment lines (“—lwidth,” default: 1) or make the resulting .PNG file transparent (“—transparent”).

If paths to BED files were provided as an input, the user can specify the “—show-annot” option to add annotations for each sequence. Annotations are visualized on the side of the matrix as rectangles spanning the corresponding start and end positions, and an annotation unit is colored in black until a color is specified in the BED file. Breakpoint lines corresponding to positions of alignment starts and ends can be added via the “—show-bp” option. The color, the line width, and the minimum length of alignments used for their visualization can be customized using the “—bp-color” (default: #7F7F7F), “—bp-lwidth” (default: 0.2), and “—bp-min-len” (default: 10 kbp) options, respectively.

**Figure 1.**
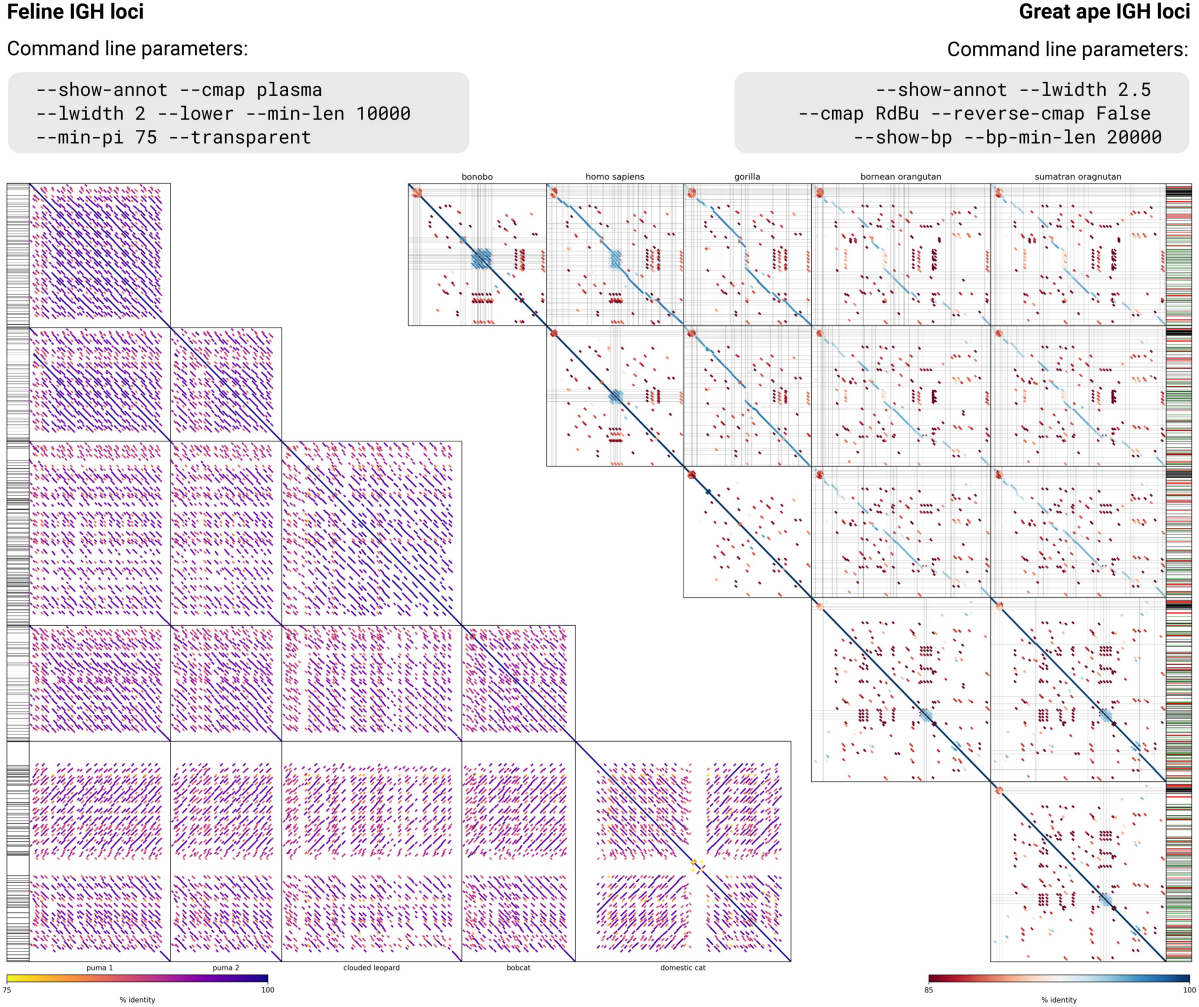
**Illustration of PatchWorkPlot work**. The left plot shows a lower triangular matrix representing alignments of five immunoglobulin heavy chain loci of four feline species (the puma haplotype 1: GCA_028749985.3; the puma haplotype 2: GCA_028749965.3, the clouded leopard: GCA_028018385.1; the bobcat: GCF_022079265.1; the domestic cat: GCA_013340865.1). The right plot shows an upper triangular matrix representing alignments of five immunoglobulin heavy chain loci of great ape species (the bonobo: GCA_029289425.3; the human: T2T-CHM13; the gorilla: GCA_029281585.3; the Bornean orangutan: GCA_028885625.3; the Sumatran orangutan: GCA_028885655.3). Command line parameters on the top of each plot were added manually for illustration purposes. Plots and legends on the bottom were generated by the tool without manual modifications.

Fig. 1 shows examples of PatchWorkPlot outputs. The lower triangular plot (Fig. 1A) shows alignments of five immunoglobulin (IG) heavy chain loci of feline species (two haplotypes of the puma, the clouded leopard, the bobcat, and the domestic cat) with lengths ranging from 1.2 to 2.3 Mbp, and the upper triangular plot (Fig. 1B) shows alignments of five IG heavy chain loci of great apes (the bonobo, the human, the gorilla, the Bornean orangutan, the Sumatran orangutan) with lengths 1.0–1.4 Mbp. The right plot shows breakpoint lines for alignments longer than 20 kbp. Both plots show positions of IG genes computed using IgDetective (Sirupurapu *et al.* 2022) in black, the right plot also shows positions of Alu and LINE/L1 repeats computed using RepeatMasker (Smit *et al.* 2013) in green and red, respectively. Both PatchWorkPlot examples illustrate how the tool allows one to visually assess the structure of segmental duplications within and across the species and compare them with positions of important genetic elements.

## 3 Results

PatchWorkPlot is designed to visualize complex genomic regions with a high density of structural variations. The tool allows the user to fine-tune visualization parameters and add annotations, enabling visual assessment of correspondence between annotated genetic elements and structural variations. PatchWorkPlot is implemented in Python 3 and requires little installation effort.

The overall performance of PatchWorkPlot depends on the performance of the chosen alignment tool and the complexity of input sequences. On average, the visualization step takes 1% of the total running time. For example, the running time on highly repetitive feline IG heavy chain loci (Fig. 1A; 15 pairwise alignments) was 45 minutes 43 seconds including alignment using LastZ, and 30 seconds using previously computed alignments (8-core CPU, 16 Gb RAM). The running time on less repetitive great ape IG heavy chain loci (Fig. 1B; 15 pairwise alignments) was 8 minutes 49 seconds (6 seconds) with (without) LastZ alignment on the same machine.

Two more test cases that include longer sequences allow one to assess the scalability of PatchWorkPlot: the inverted region in human 8p23 (Antonacci *et al.* 2009) and the centromere of human chromosome 5. The inversion in 8p23 was extracted from three human genomes (human T2T GCF_009914755.1, human HG002 maternal GCA_018852615.3, human HG002 paternal GCA_018852605.3) and aligned to the chimpanzee genome (mPanTro3 GCF_028858775.2) and the gorilla genome (mGorGor2.1 GCF_029281585.2) to find corresponding orthologous regions. Lengths of the selected regions range from 8.04 to 10.91 Mbp, their coordinates are provided in Table S1, available as supplementary data at *Bioinformatics* online. 9501 alignments were computed using minimap2 for all pairs of input sequences and visualized using PatchWorkPlot with default parameters (Fig. 1, available as supplementary data at *Bioinformatics* online). The visualization time was 17 seconds on a 128-core CPU server with 2.2 Tb RAM. The human chromosome 5 centromere (2.93 Mbp) was aligned against centromeres of orthologous chromosomes of chimpanzee and gorilla (2.19 and 5.68 Mbp, respectively) using minimap2 and resulted in 21 947 479 short alignments (Table S1 and Fig. 2, available as supplementary data at *Bioinformatics* online). The visualization time of alignments exceeding 120 nt was 50 832 seconds (∼14 hours) on the same server. These test cases showed that the repetitiveness of input sequences determines the number of input alignments and thus the running time of PatchWorkPlot.

## Supplementary Material

btaf504_Supplementary_Data

## Data Availability

The data underlying this article are available in NCBI at https://www.ncbi.nlm.nih.gov/datasets/genome/, and can be accessed with the following accession numbers: GCA_028749985.3 (puma, haplotype 1), GCA_028749965.3 (puma, haplotype 2), GCA_028018385.1 (clouded leopard), GCF_022079265.1 (bobcat), GCA_013340865.1 (domestic cat), GCA_029289425.3 (bonobo), GCA_009914755.4 (human, T2T-CHM13), GCA_018852615.3 (human, HG002 maternal haplotype), GCA_018852605.3 (human, HG002 paternal haplotype), GCA_029281585.3 (gorilla), GCA_028885625.3 (Bornean orangutan), GCA_028885655.3 (Sumatran orangutan), and GCF_028858775.2 (chimpanzee).
