# Supplementary material for: PatchWorkPlot: simultaneous visualization of local alignments across multiple sequences
Source: Bioinformatics. 2025 Sep 15;41(10):btaf504. doi: 10.1093/bioinformatics/btaf504 (PMC12516306; doi:10.1093/bioinformatics/btaf504)
Supplement: btaf504_Supplementary_Data [file btaf504_supplementary_data.pdf]

PatchWorkPlot: simultaneous visualization of local alignments across multiple sequences. Supplemental Materials

Mariia Pospelova[1] & Yana Safonova[1,2,*]

[1] Computer Science and Engineering Department, Pennsylvania State University, State College, PA 16802, USA
[2] Huck Institutes of Life Science, Pennsylvania State University, State College, PA 16802, USA
[*] Corresponding author: yana@psu.edu

Region	Species	Accession number	Contig and coordinates	Chr
Chromosome 5 centromere	human (T2T)	GCF_009914755.1	NC_060929, 46,664,500 – 49,598,300	5
	chimpanzee	GCF_028858775.2	NC_072402.2, 64,391,200 – 66,584,700	4
	gorilla	GCF_029281585.2	NC_073243.2, 59,933,200 – 65,608,600	19
Complex inversion in 8p23.1	human (T2T)	GCF_009914755.1	NC_060932.1, 5,594,000-13,720,000	8
	human (HG002 maternal)	GCA_018852615.3	CP139513.2, 5,810,000-13,850,000	8
	human (HG002 paternal)	GCA_018852605.3	CP139536.2, 5,690,000-13,850,000	8
	chimpanzee	GCF_028858775.2	NC_072405.2, 11,190,000-22,100,000	7
	gorilla	GCF_029281585.2	NC_073231.2, 9,910,000-18,150,000	7

Table S1. Coordinates of the human chromosome 5 centromere and a complex inversion in human 8p23 and regions orthologous to them in chimpanzee and gorilla genomes.

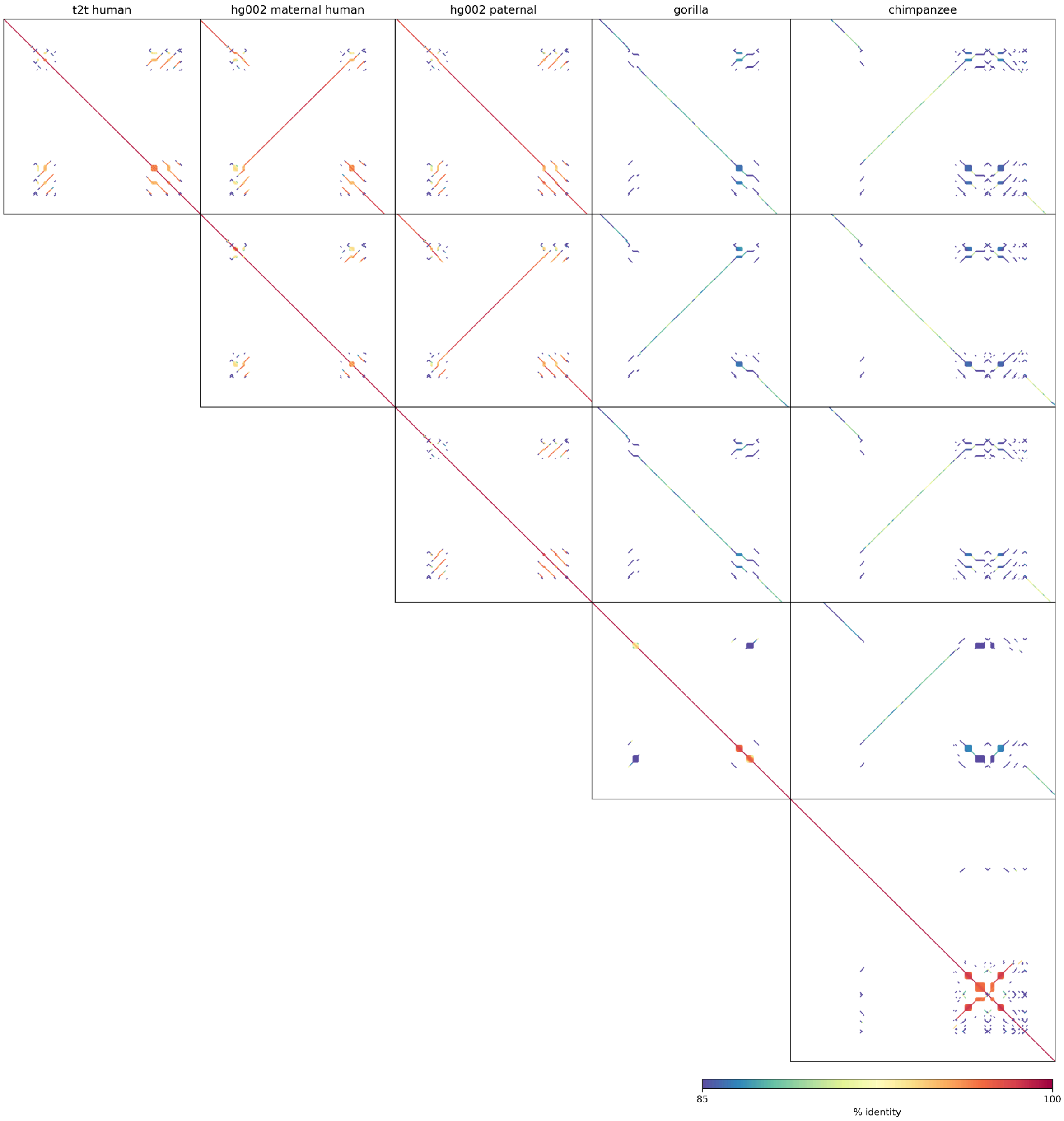

Figure S1. Example of PatchWorkPlot output on a complex inversion in human 8p23.1 using the human T2T genome and two haplotypes of HG002 and orthologous regions in chimpanzee and gorilla. Coordinates of the aligned regions are provided in **Table S1**. The regions were aligned using minimap2 with parameters used by PatchWorkPlot by default ("--secondary=yes -P -k 10 -w 5 --no-long-join -r 100 -g 50"). Default parameters of PatchWorkPlot were used.

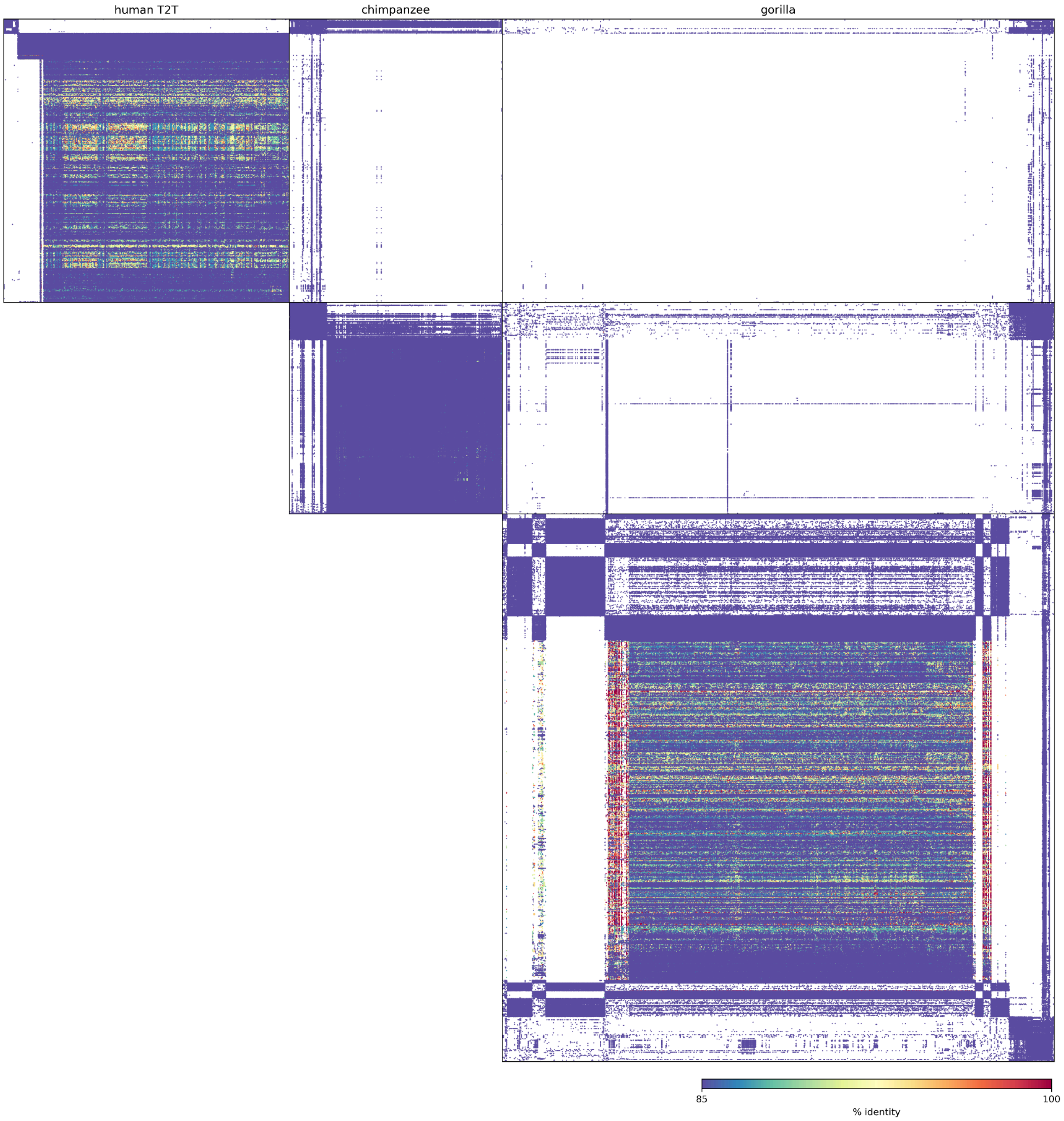

Figure S2. Example of PatchWorkPlot output on the human chromosome 5 centromere, chimpanzee chromosome 4 centromere, and gorilla chromosome 19 centromere. Coordinates of the centromere regions are provided in Table S1. Centromeres were aligned using minimap2 with parameters used by PatchWorkPlot by default (“--secondary=yes -P -k 10 -w 5 --no-long-join -r 100 -g 50”). The only non-default PatchWorkPlot parameter was “`--min-len 120`”.
